# HDAC5 Inhibition as a Therapeutic Strategy for Titin Deficiency-Induced Cardiac Remodeling: Insights from Human iPSC Models

**DOI:** 10.3390/medicines12040026

**Published:** 2025-10-27

**Authors:** Arif Ul Hasan, Sachiko Sato, Mami Obara, Yukiko Kondo, Eiichi Taira

**Affiliations:** Department of Pharmacology, School of Medicine, Iwate Medical University, Iwate 028-3694, Japan

**Keywords:** cardiomyopathy, epigenetic therapy, histone deacetylase, induced pluripotent cells

## Abstract

Background/Objectives: Dilated cardiomyopathy (DCM) is a prevalent and life-threatening heart muscle disease often caused by titin (*TTN*) truncating variants (*TTN*tv). While *TTN*tvs are the most common genetic cause of heritable DCM, the precise downstream regulatory mechanisms linking *TTN* deficiency to cardiac dysfunction and maladaptive fibrotic remodeling remain incompletely understood. This study aimed to identify key epigenetic regulators of *TTN*-mediated gene expression and explore their potential as therapeutic targets, utilizing human patient data and in vitro models. Methods: We analyzed RNA sequencing (RNA-seq) data from left ventricles of non-failing donors and cardiomyopathy patients (DCM, HCM, PPCM) (GSE141910). To model *TTN* deficiency, we silenced *TTN* in human iPSC-derived cardiomyocytes (iPSC-CMs) and evaluated changes in cardiac function genes (*MYH6*, *NPPA*) and fibrosis-associated genes (*COL1A1*, *COL3A1*, *COL14A1*). We further tested the effects of TMP-195, a class IIa histone deacetylase (HDAC) inhibitor, and individual knockdowns of HDAC4/5/7/9. Results: In both human patient data and the *TTN* knockdown iPSC-CM model, *TTN* deficiency suppressed *MYH6* and *NPPA* while upregulating fibrosis-associated genes. Treatment with TMP-195 restored NPPA and MYH6 expression and suppressed collagen genes, without altering *TTN* expression. Among the HDACs tested, HDAC5 knockdown was most consistently associated with improved cardiac markers and reduced fibrotic gene expression. Co-silencing *TTN* and *HDAC5* replicated these beneficial effects. Furthermore, the administration of TMP-195 enhanced the modulation of *NPPA* and *COL1A1*, though its impact on *COL3A1* and *COL14A1* was not similarly enhanced. Conclusions: Our findings identify HDAC5 as a key epigenetic regulator of maladaptive gene expression in *TTN* deficiency. Although the precise mechanisms remain to be clarified, the ability of pharmacological HDAC5 inhibition with TMP-195 to reverse *TTN*-deficiency-induced gene dysregulation highlights its promising translational potential for *TTN*-related cardiomyopathies.

## 1. Introduction

Cardiomyopathies are a group of complex and diverse heart muscle diseases that pose a significant global health burden [1]. They are broadly classified into several types, including dilated cardiomyopathy (DCM), hypertrophic cardiomyopathy (HCM), restrictive cardiomyopathy (RCM), and arrhythmogenic cardiomyopathy (AC), each with distinct clinical presentations and pathological mechanisms [2,3]. Among these, DCM is the most common form, characterized by left ventricular dilatation and systolic dysfunction, often leading to heart failure and arrhythmias [1]. Genetic factors play a crucial role in the etiology of many cardiomyopathies, with approximately 30–50% of DCM cases having a familial origin [4,5].

The titin gene (*TTN*), which encodes the giant sarcomeric protein titin, is increasingly recognized as a key genetic determinant in various cardiomyopathies [6]. Titin is a crucial structural and mechanosensory protein within the cardiac sarcomere, responsible for maintaining sarcomere integrity, providing passive elasticity, and actively transmitting force [6,7]. Truncating variants in *TTN* (*TTNtvs*) are the most common genetic cause of heritable DCM [5,7]. While *TTNtvs* are highly prevalent in DCM cohorts, their penetrance for the disease in the general population can be low, posing a challenge for clinical interpretation due to the gene’s immense size and extensive genetic variation [8]. The precise mechanisms by which *TTN* truncations lead to DCM are still debated, with theories including haploinsufficiency (reduced functional protein) and a “poison peptide” effect (interference by truncated proteins) [9,10]. Beyond DCM, *TTN* variants have also been implicated in other cardiomyopathies such as HCM [11], peripartum cardiomyopathy (PPCM) [12], and arrhythmogenic right ventricular cardiomyopathy (ARVC) [13].

Cardiac remodeling, characterized by maladaptive changes in myocardial structure and function, is a hallmark of cardiomyopathy progression [14,15]. A critical component of this remodeling is myocardial fibrosis, which involves the excessive deposition of extracellular matrix (ECM) proteins, primarily collagen I and III [11,16]. Fibrosis contributes to cardiac dysfunction by stiffening the myocardial matrix, impairing electrical conduction, and ultimately leading to heart failure [1]. Current treatments for myocardial fibrosis can alleviate symptoms but often do not completely reverse the established scarring, highlighting the need for novel therapeutic targets [16,17]. The reversibility and variability of epigenetic modifications make them a compelling area for therapeutic intervention in cardiac diseases [17,18].

Epigenetic mechanisms, including DNA methylation, histone modifications (such as acetylation and deacetylation), and non-coding RNAs, regulate gene expression without altering the underlying DNA sequence [18,19]. Among them, histone deacetylases (HDACs) are enzymes that remove acetyl groups from histones, generally leading to transcriptional repression, and are implicated in cardiovascular diseases such as cardiac hypertrophy and fibrosis [19]. Of particular interest, HDAC5, a class II HDAC, has been associated with pathological cardiac remodeling [20]. Previous studies indicate that HDAC5 represses cardiac functional genes and promotes fibrosis, while its inhibition can reverse these effects [21,22]. More broadly, Class II HDACs (HDAC4, 5, 7, 9) act as signal-responsive repressors of myocyte enhancer factor 2 (MEF2), a transcription factor essential for cardiac development, morphogenesis, and remodeling [23]. Loss of MEF2, particularly MEF2C, is detrimental: global *Mef2c* disruption in mice causes embryonic lethality, severe cardiovascular malformations, and downregulation of cardiomyocyte genes including *Ttn*, *Tnnt2*, and *Nppa* [24,25]. Together, these observations highlight MEF2C as a central regulator of cardiac gene networks and suggest that its upstream modulators, including HDAC5, may exert profound effects on disease progression.

The pivotal role of *TTN* in cardiomyopathies, together with the emerging importance of epigenetic regulation, raises the possibility that the interplay between *TTN* and HDAC5 could provide new insights into disease pathogenesis. Building on this rationale, in this study, we examine *TTN* expression across cardiomyopathies (DCM, HCM, PPCM), with a focus on its downregulation in DCM patients. Using human iPSC-derived cardiomyocytes (iPSC-CMs), we further investigate *TTN* deficiency-induced gene dysregulation and assess whether HDAC5 modulation can restore cardioprotective gene programs, highlighting its potential as a therapeutic strategy.

## 2. Materials and Methods

### 2.1. Public Dataset Acquisition and Data Processing

RNA-sequencing data from left ventricles of non-failing donors (NF, *N* = 161) and patients with DCM (*N* = 161), HCM (*N* = 28), and PPCM (*N* = 6) (GEO Accession: GSE141910) [26,27] were retrieved and analyzed using both model-based and non-parametric approaches. Primary differential expression analysis was performed using the GEO2R, which estimates gene-wise dispersion and normalizes counts to model differences between groups while accounting for imbalanced group sizes. Pairwise contrasts were specified for DCM vs. NF and HCM vs. NF. Results were reported as log2 fold-change values with Benjamini–Hochberg-adjusted *p*-values, with a significance threshold of 0.05 as integrated into the GEO2R pipeline (https://www.ncbi.nlm.nih.gov/geo/query/acc.cgi?acc=GSE141910, accessed on 12 June 2025). Data were downloaded and further analyzed in GraphPad Prism 9.0.0 (GraphPad Software, Boston, MA, USA). A volcano plot was generated using GraphPad Prism to visualize differential expression patterns. To validate robustness, selected differentially expressed genes were reanalyzed using the non-parametric Kruskal–Wallis test followed by Dunn’s post hoc test for multiple comparisons. This complementary strategy ensured consistency across both parametric and non-parametric frameworks, particularly given the unequal sample sizes among groups. To assess the expression MEF2 isoforms in cardiomyocytes, we retrieved a consensus dataset from the Human Protein Atlas (HPA) and GTEx transcriptomics, which provided normalized transcripts per million (nTPM) values (source: Human Protein Atlas; https://www.proteinatlas.org/, accessed on 12 June 2025) [28].

### 2.2. Cell Lines, Culture and Reagents for Major Interventions

The human iPSC line, HPS4290 201B7-Ff was purchased from the RIKEN Bioresource Research Center, Japan. Cells were plated and maintained in StemFlex Medium Kit (A3349401, Thermo Fisher Scientific, Grand Island, NY, USA) according to the manufacturer’s instructions. Briefly, 12-well plates were coated with 0.5 µg/cm^2^ iMatryx-511 (892011, Nippi Incorporated, Tokyo, Japan), diluted in DPBS (14190-144, Thermo Fisher Scientific, Fountain Drive, Paisley, UK) and incubated at 37 °C for 1 h. All cultures were maintained at 37 °C in a humidified atmosphere of 95% air and 5% CO_2_, and they were passaged at 50–60% confluency. Cells were detached using TrypLE Select (12563-011, Thermo Fisher Scientific, Grand Island, NY, USA), and the culture medium (StemFlex) was supplemented with 1x RevitaCell Supplement (A2644501, Thermo Fisher Scientific, Grand Island, NY, USA). The medium was refreshed the next day and subsequently every other day (StemFlex only).

When the cells reached 70% confluency, cardiomyocyte differentiation was induced using the PSC Cardiomyocyte Differentiation Kit (A29212-01, Thermo Fisher Scientific, Grand Island, NY, USA) following the manufacturer’s protocol. Differentiation was initiated with Cardiomyocyte Differentiation Medium A, replaced after 2 days with Cardiomyocyte Differentiation Medium B. After another 2 days and onwards, the cells were maintained in Cardiomyocyte Maintenance Medium with medium changes every other day. Chemical interventions were performed between days 9 and 10, coinciding with the onset of spontaneous beating. For treatment, cells were exposed to TMP-195 (23242, Cayman Chemical, AnnArbor, MI, USA), while control groups received an equivalent volume of dimethyl sulfoxide (DMSO; 13408-64, Nacalai Tesque, Kyoto, Japan) for 24 h.

### 2.3. Gene Silencing Using siRNA Transfection

Gene silencing was performed as previously described [29]. Briefly, 2000 cells were seeded in 24-well plates with 500 µL of medium. Once colonies reached approximately 70% confluency, the StemFlex medium was replaced with 500 µL of Cardiomyocyte Differentiation Medium A, and cells were transfected with 50 nM siRNA targeting *TTN* (HSS141050), *HDAC4* (HSS114674), *HDAC5* (HSS145409), *HDAC7* (HSS147499), *HDAC9* (HSS190582) (all from Thermo Fisher Scientific, Waltham, MA, USA), or with a nonspecific negative control siRNA (Medium GC Duplex #2, 465372, Thermo Fisher Scientific, Waltham, MA, USA). Transfections were carried out using 1.5 µL of Lipofectamine RNAiMAX reagent (13778, Thermo Fisher Scientific, Carlsbad, CA, USA) diluted in 100 µL Opti-MEM I Reduced Serum Medium (31985062, Thermo Fisher Scientific, Grand Island, NY, USA). After 48 h, the medium was replaced with Cardiomyocyte Differentiation Medium B, and differentiation was continued as described above.

### 2.4. Total RNA Extraction, cDNA Synthesis, and Quantitative Real-Time PCR (qRT PCR)

Total RNA was extracted, and qRT-PCR was performed as previously described [30,31]. RNA isolation was carried out using TRIzol reagent (15596018, Thermo Fisher Scientific, Carlsbad, CA, USA) according to the manufacturer’s instructions. First-strand cDNA was synthesized from 500 ng RNA using the PrimeScript RT reagent Kit (RR037A, Takara Bio, Shiga, Japan). Quantitative PCR was performed on a 7500 Fast Real-Time PCR System (Applied Biosystems, Tokyo, Japan) using PowerTrack SYBR Green Master Mix (A46109, Thermo Fisher Scientific, Vilnius, LT, USA). The final primer concentration was 250 nM in a 20 µL reaction volume. Cycling conditions were: 95 °C for 20 s, followed by 40 cycles of 95 °C for 3 s and 60 °C for 30 s. A dissociation curve was performed with default settings. For each gene, relative expression levels were normalized to control (set as 1.0). Data represent the mean of three biological replicates. Primer sequences are provided in Table 1.

### 2.5. Statistical Analysis

All statistical analyses were performed using GraphPad Prism 9.0.0 (GraphPad Software, Boston, MA, USA). For RNA-seq data, GEO2R was used with default parameters as described above. Additionally, the non-parametric Kruskal–Wallis test was applied to assess the gene expression differences among groups. For two-group comparisons, two-tailed unpaired Student’s *t*-tests were used. For multiple groups, one-way ANOVA followed by Tukey’s post hoc test was applied. A *p*-value of less than 0.05 was considered statistically significant. Error bars in graphs represent the standard error of the mean (SEM).

## 3. Results

### 3.1. TTN and Associated Gene Expression Alterations in Human Cardiomyopathies

**Figure 1:**
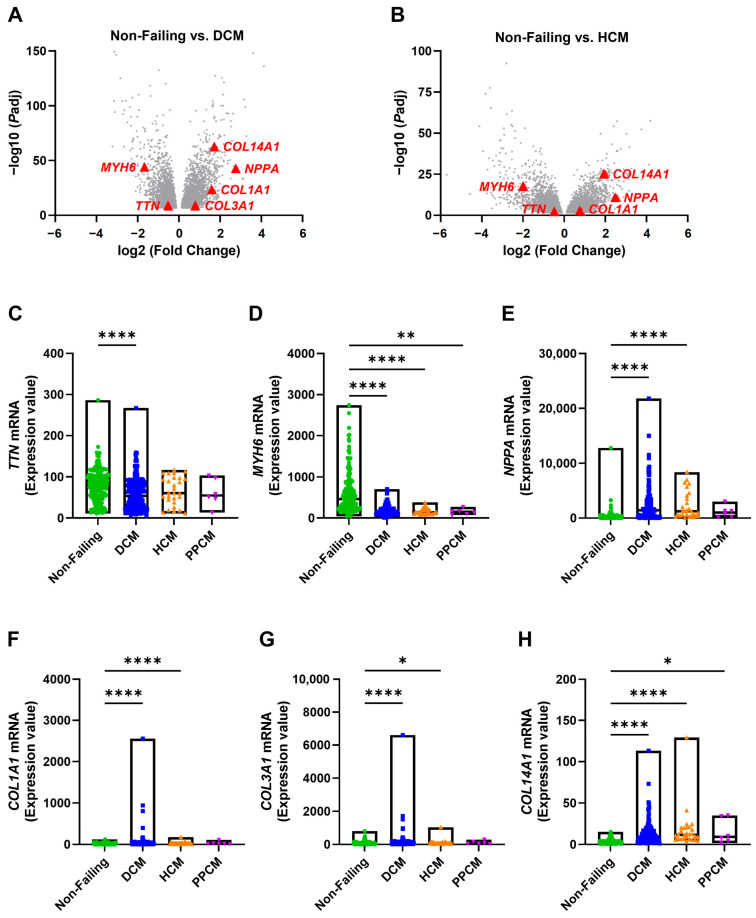
*TTN* and cardiac function- and fibrosis-associated gene expression are altered in human cardiomyopathy subtypes. RNA-sequencing data from the left ventricles of non-failing donors (Non-Failing, *N* = 161) and patients with dilated cardiomyopathy (DCM, *N* = 161), hypertrophic cardiomyopathy (HCM, *N* = 28), and peripartum cardiomyopathy (PPCM, *N* = 6) were obtained from the Gene Expression Omnibus (GEO Accession: GSE141910). Primary analysis was performed using the GEO2R portal, and results are shown as normalized counts, with log2 fold-change values and Benjamini–Hochberg adjusted *p*-values. (**A**,**B**) Volcano plots showing significantly differentially expressed genes between non-failing vs. DCM and non-failing vs. HCM samples. (**C**–**H**) mRNA expression levels of selected genes compared across groups. Each dot represents an individual sample; floating bars indicate minimum to maximum values, and horizontal lines represent medians. Statistical analysis: Panels (**C**–**H**) were analyzed using the non-parametric Kruskal–Wallis test followed by Dunn’s post hoc test. * *p* < 0.05; ** *p* < 0.01; **** *p* < 0.0001.

To investigate gene expression alterations in heart failure, we retrieved RNA-sequencing data from left ventricles of non-failing donors (NF, *N* = 161) and patients with DCM (*N* = 161), HCM (*N* = 28), and PPCM (*N* = 6) available at the Gene Expression Omnibus (GEO; Accession: GSE141910) [26,27]. Primary differential expression analysis was performed using the GEO2R, with pairwise contrasts specified for DCM vs. NF and HCM vs. NF. Because of the very small sample size, PPCM vs. NF was not included in this analysis. This analysis suggested that compared to non-failing controls, *TTN* expression was significantly downregulated in both DCM and HCM patients. The contractile gene *MYH6* was also downregulated, whereas the stress-responsive marker *NPPA* was upregulated across both groups (Figure 1A,B). In addition, while fibrosis-related genes *COL1A1* and *COL14A1* were upregulated in both groups, *COL3A1* was upregulated only in DCM (Figure 1A,B). To ensure robustness, we complemented the GEO2R-based analysis with non-parametric Kruskal–Wallis testing across all four groups. This approach yielded consistent results despite the unbalanced sample sizes but indicated that *TTN* expression was significantly downregulated only in DCM, not in HCM (Figure 1C). *MYH6* was downregulated across all three cardiomyopathy groups, while *NPPA* was upregulated in DCM and HCM but not significantly in PPCM (Figure 1D,E). Moreover, *COL1A1*, *COL3A1*, and *COL14A1* were predominantly upregulated in DCM and HCM patients (Figure 1F–H). The discrepancy regarding *TTN* and *COL3A1* between differential expression and Kruskal–Wallis testing most likely reflects unbalanced sample sizes among groups but does not interfere with our core conclusions.

### 3.2. TTN Deficiency Suppresses Cardiac Functional Genes and Augments Fibrosis-Associated Genes in iPSC-CMs

**Figure 2:**
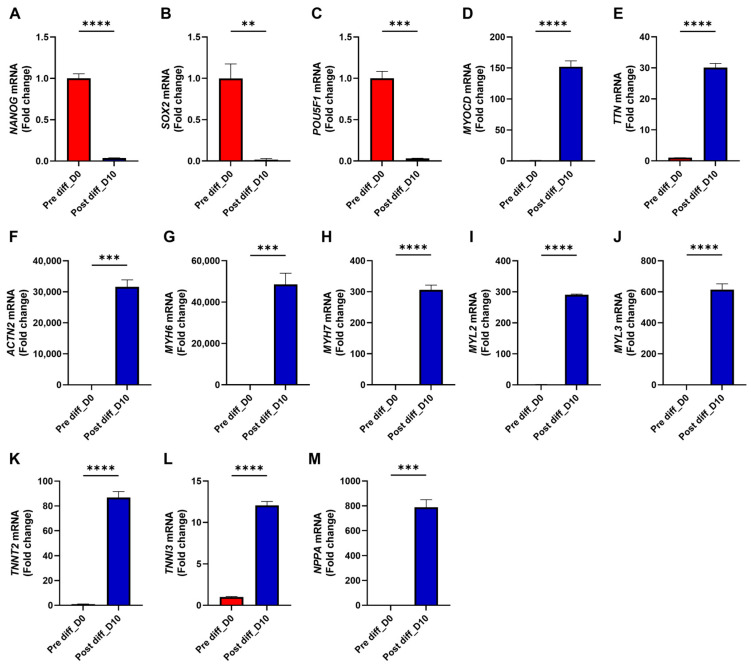
Cardiogenic induction of iPSCs successfully differentiates them into functional cardiomyocytes. Human iPSCs were induced to differentiate into cardiomyocytes for 10 days as described in Materials and Methods. mRNA was extracted from cells before differentiation (Pre-diff_D0) and at day 10 of differentiation (Post-diff_D10). (**A**–**M**) mRNA expression levels of target genes were quantified by qRT-PCR. Each condition was tested in three biological replicates (*n* = 3). Statistical analysis: All panels were analyzed using two-tailed unpaired Student’s *t*-tests. ** *p* < 0.01; *** *p* < 0.001; **** *p* < 0.0001. Bars represent mean + SEM.

To establish an in vitro system, we differentiated human iPS cells into cardiomyocytes using a defined protocol (see Section 2). RT-PCR analysis showed marked downregulation of pluripotency markers (*NANOG*, *SOX2*, and *POU5F1*), confirming exit from pluripotency (Figure 2A–C). In parallel, the cardiac transcriptional regulator *MYOCD* was strongly induced, accompanied by robust expression of sarcomeric structural genes (*TTN* and *ACTN2*), myosin heavy chains (*MYH6* and *MYH7*), myosin light chains (*MYL2* and *MYL3*), and thin filament regulators (*TNNT2* and *TNNI3*) (Figure 2D–L). The chamber-specific marker *NPPA* was also significantly upregulated (Figure 2M), together supporting successful and efficient differentiation of iPS cells into cardiomyocytes.

**Figure 3:**
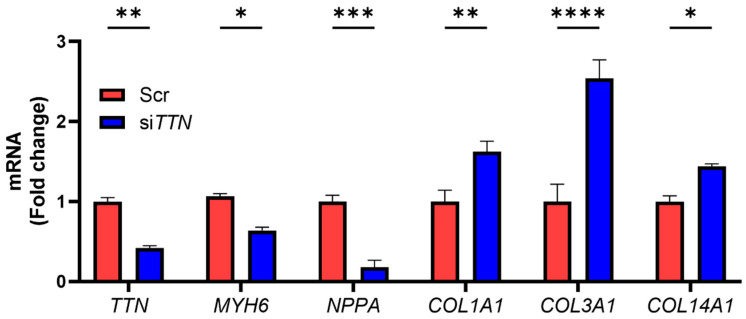
*TTN* deficiency regulates cardiac function- and fibrosis-associated gene expression. Human iPSC-derived cardiomyocytes were transfected with scrambled control siRNA (Scr) or *TTN*-specific siRNA (si*TTN*), and mRNA expression levels of target genes were quantified by qRT-PCR. Each condition was tested in three biological replicates (*n* = 3). Statistical analysis: Each gene was analyzed using two-tailed unpaired Student’s *t*-tests. * *p* < 0.05; ** *p* < 0.01; *** *p* < 0.001; **** *p* < 0.0001. Bars represent mean + SEM.

To directly examine the role of *TTN*, we silenced *TTN* in iPSC-CMs. *TTN* knockdown led to downregulation of *MYH6*, while upregulating *COL1A1*, *COL3A1*, and *COL14A1* (Figure 3). As expected, *NPPA* expression was also reduced, likely reflecting its dependence on mechanical stimuli such as pressure overload. These findings suggest that *TTN* acts as an upstream regulator of both cardiac contractile and fibrosis-associated gene expression, and its deficiency induces a dual phenotype, characterized by suppressed functional genes (e.g., *MYH6*, *NPPA*) and upregulated fibrotic genes (e.g., *COL1A1*, *COL3A1*, *COL14A1*).

### 3.3. Class II HDAC Inhibitor TMP-195 Restores Cardiac Functional Genes and Suppresses Fibrosis-Associated Genes

**Figure 4:**
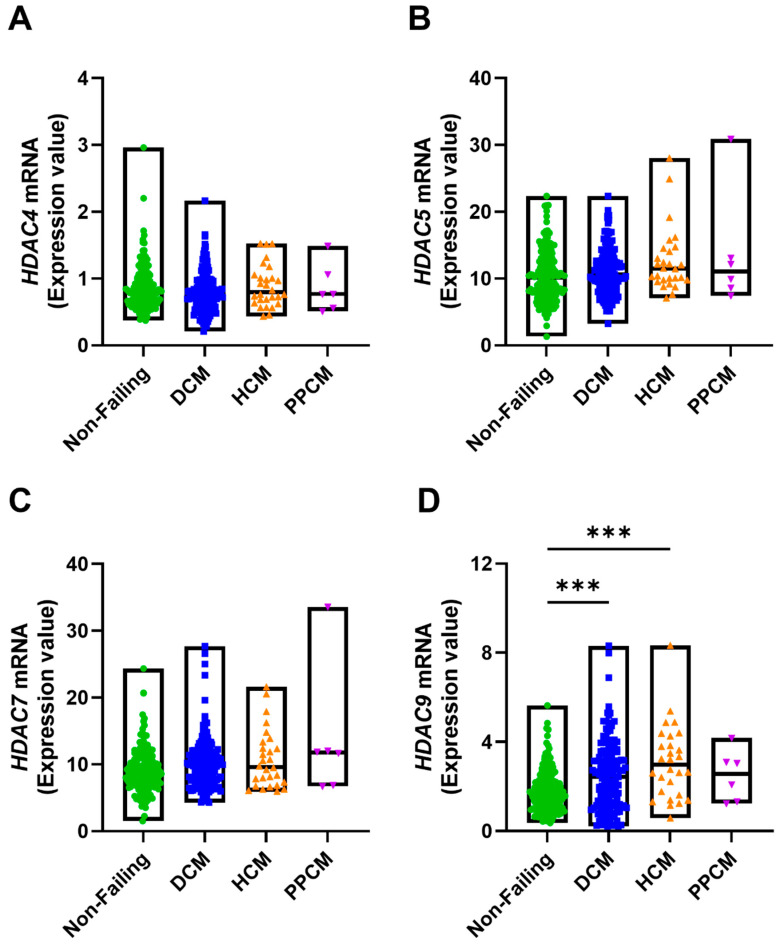
Class II HDACs are differentially expressed in dilated cardiomyopathy patients. RNA-sequencing data from the left ventricles of non-failing donors (N = 161) and patients with DCM (N = 161), HCM (N = 28), and PPCM (N = 6) were obtained from the Gene Expression Omnibus (GEO Accession: GSE141910). Primary analysis was performed using the GEO2R portal, and results are shown as normalized counts, with log2 fold-change values and Benjamini–Hochberg-adjusted *p*-values. (**A**–**D**) mRNA expression levels of selected genes were compared across groups. Each dot represents an individual sample; floating bars indicate minimum to maximum values, and horizontal lines represent medians. Statistical analysis: Panels were analyzed using the non-parametric Kruskal–Wallis test followed by Dunn’s post hoc test. *** *p* < 0.001.

To investigate the involvement of Class II HDACs in cardiomyopathy, we analyzed RNA-sequencing data from left ventricular samples of patients with DCM, HCM and PPCM (GEO Accession: GSE141910). However, HDAC9 expression was increased in DCM and HCM, and other Class II HDACs (HDAC4, 5, 7) were not significantly changed (Figure 4A–D). This finding indicates that while some Class II HDACs may act as effectors in DCM and HCM, others may function as upstream regulators or show context-dependent regulation. This possibility was addressed in subsequent experiments.

**Figure 5:**
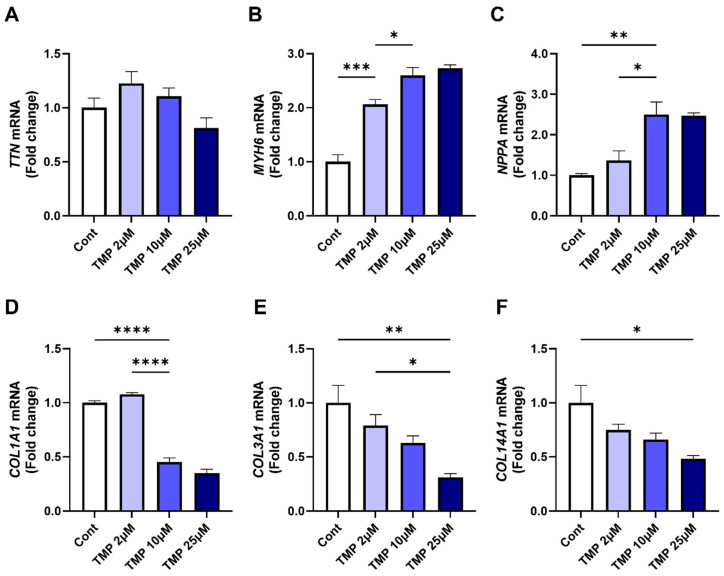
Class II HDAC inhibitor TMP-195 enhances expression of cardiac functional genes and suppresses fibrosis-associated gene expression in cardiomyocytes. Human iPSC-derived cardiomyocytes were treated with DMSO (Cont) or TMP-195 at concentrations of 2, 10, or 25 μM. (**A**–**F**) mRNA expression levels of target genes were quantified by qRT-PCR. Each condition was tested in three biological replicates (n = 3). Statistical analysis: All panels were analyzed using one-way ANOVA followed by Tukey’s post hoc test. * *p* < 0.05; ** *p* < 0.01; *** *p* < 0.001; **** *p* < 0.0001. Bars represent mean + SEM.

We next examined the functional effects of pharmacological Class II HDAC inhibition in iPSC-CMs. Treatment with increasing doses of TMP-195, a broad-spectrum Class II HDAC inhibitor, for 24 h had no effect on *TTN* expression but induced a dose-dependent upregulation of cardiac functional genes *MYH6* and *NPPA* (Figure 5A–C). Conversely, TMP-195 treatment significantly downregulated fibrosis-associated genes, including *COL1A1*, *COL3A1*, and *COL14A1* (Figure 5D–F). Together, these findings demonstrate that Class II HDAC inhibition by TMP-195 enhances protective cardiac gene expression while selectively suppressing profibrotic signaling. They further suggest that pharmacological targeting of Class II HDACs may represent a therapeutic strategy for mitigating *TTN* deficiency-related gene dysregulation in cardiomyopathy.

### 3.4. TMP-195 Restores Cardiac Functional Gene Expression and Suppresses Fibrosis-Associated Gene Following TTN Silencing

**Figure 6:**
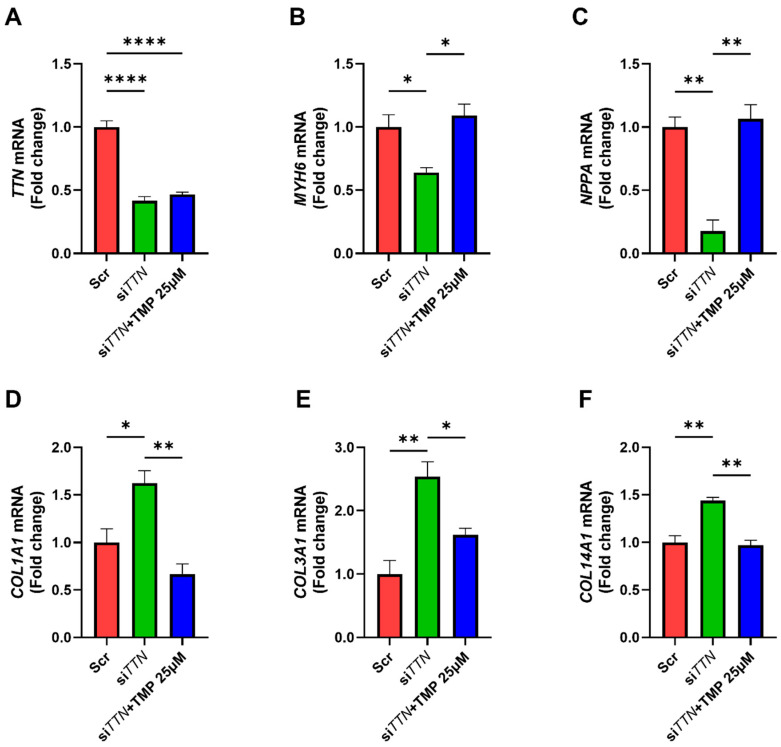
TMP-195 restores cardiac functional gene expression and suppresses fibrosis-associated gene expression following *TTN* silencing. Human iPSC-derived cardiomyocytes were transfected with scrambled control siRNA (Scr) or *TTN*-specific siRNA (si*TTN*), followed by treatment with 25 μM TMP-195. (**A**–**F**) mRNA expression levels of target genes were quantified by qRT-PCR. Each condition was tested in three biological replicates (n = 3). Statistical analysis: All panels were analyzed using one-way ANOVA followed by Tukey’s post hoc test. * *p* < 0.05; ** *p* < 0.01; **** *p* < 0.0001. Bars represent mean + SEM.

We next investigated whether TMP-195 could reverse gene expression changes induced by *TTN* silencing in iPSC-CMs. While TMP-195 did not alter *TTN* levels (Figure 6A), it successfully restored the expression of *MYH6* (Figure 6B). Interestingly, while *NPPA* was upregulated in DCM patient samples, it was reduced in *TTN*-silenced iPSC-CMs, likely reflecting the absence of systemic hemodynamic stress in vitro. Notably, pharmacological inhibition of Class IIa HDACs with TMP-195 restored *NPPA* expression, suggesting that *NPPA* regulation integrates both stress-dependent and epigenetic mechanisms (Figure 6C). In addition, TMP-195 significantly attenuated the upregulation of *COL1A1*, *COL3A1*, and *COL14A1* observed upon *TTN* knockdown (Figure 6D–F). These results indicate that TMP-195 mitigates *TTN*-deficiency-induced dysregulation of both functional and fibrotic genes, independent of *TTN* expression itself.

### 3.5. HDAC5 Inhibition Promotes Cardiac Functional Gene Expression and Suppresses Fibrosis-Associated Genes

**Figure 7:**
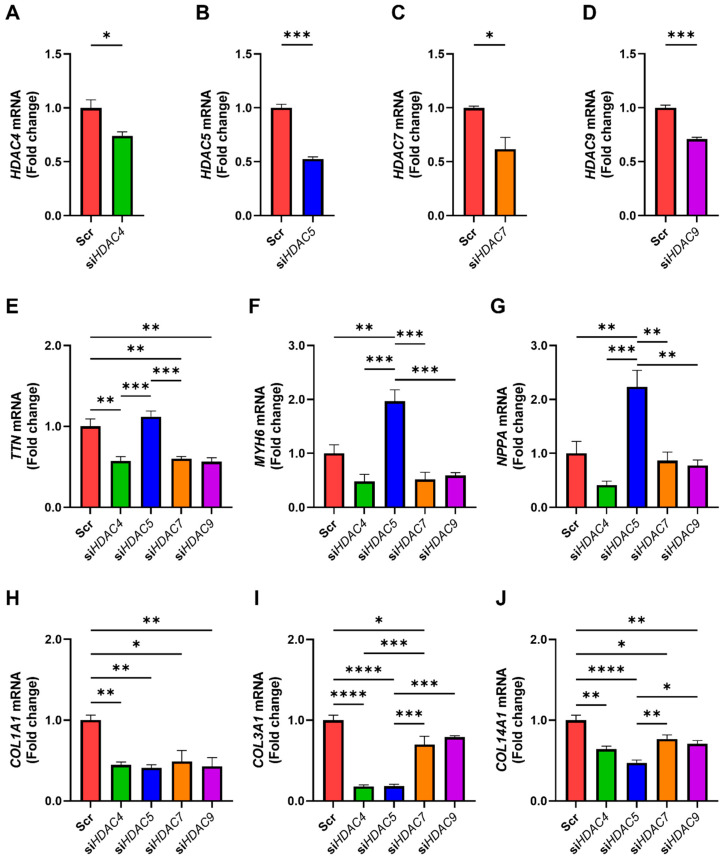
HDAC5 inhibition promotes cardiac functional gene expression and suppresses fibrosis-associated genes. Human iPSC-derived cardiomyocytes were transfected with scrambled control siRNA (Scr), or siRNAs specific for *HDAC4*, *HDAC5*, *HDAC7*, and *HDAC9* (si*HDAC4*, si*HDAC5*, si*HDAC7*, and si*HDAC9*, respectively). (**A**–**J**) mRNA expression levels of target genes were quantified by qRT-PCR. Each condition was tested in three biological replicates (*n* = 3). Statistical analysis: All panels were analyzed using one-way ANOVA followed by Tukey’s post hoc test. * *p* < 0.05; ** *p* < 0.01; *** *p* < 0.001; **** *p* < 0.0001. Bars represent mean + SEM.

Since TMP-195 targets multiple Class II HDACs, including *HDAC4*, *HDAC5*, *HDAC7*, and *HDAC9*, we aimed to identify which isoform primarily mediates the observed effects in iPSC-CMs. We individually silenced each *HDAC* in cardiomyocytes and confirmed efficient knockdown (Figure 7A–D). Silencing of *HDAC4*, *HDAC7*, or *HDAC9* led to downregulation of *TTN*, *MYH6*, and *NPPA*. In contrast, *HDAC5* knockdown had no effect on *TTN* but significantly upregulated *MYH6* and *NPPA* (Figure 7E–G). All four *HDACs*, when silenced, reduced the expression of *COL1A1*, *COL3A1*, and *COL14A1*, with *HDAC5* showing the most pronounced antifibrotic effect (Figure 7H–J). These findings highlight HDAC5 as a key epigenetic regulator that represses cardiac functional genes while promoting fibrosis, suggesting that its inhibition is a promising strategy to counteract cardiac dysfunction in *TTN*-deficient conditions.

### 3.6. TMP-195 Reverses TTN Deficiency-Induced Gene Alterations Through HDAC5 Inhibition

**Figure 8:**
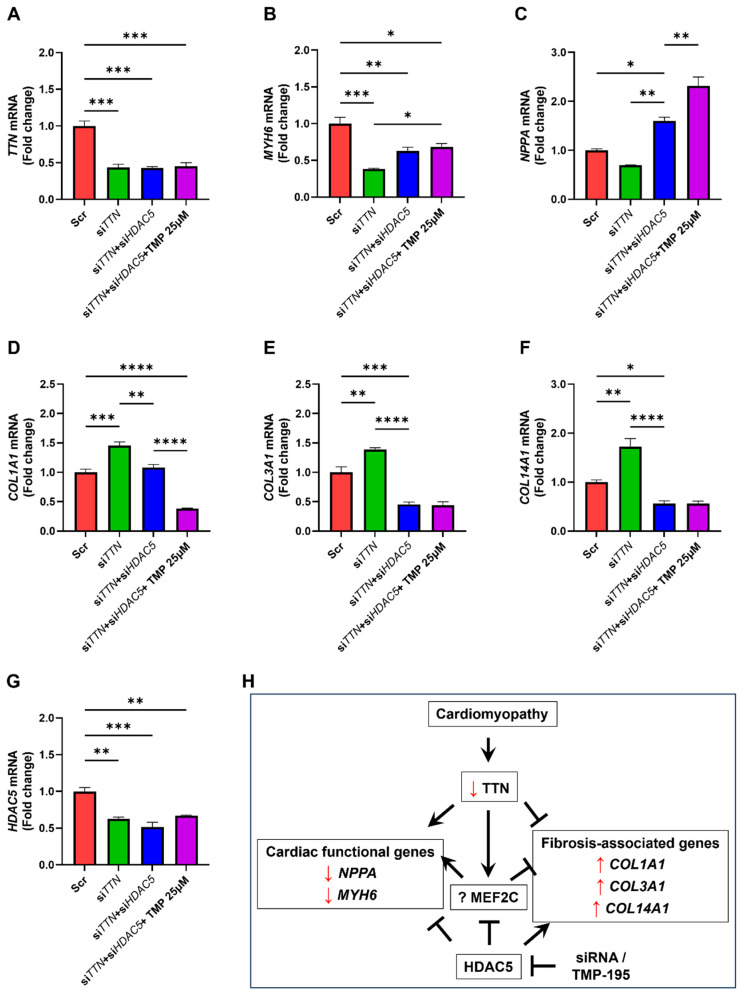
TMP-195 reverses *TTN* deficiency-induced gene alterations through HDAC5 inhibition. (**A**–**G**) Human iPSC-derived cardiomyocytes were transfected with scrambled control siRNA (Scr), *TTN*-specific siRNA (si*TTN*), or a combination of *TTN* and *HDAC5* siRNAs (si*TTN* + si*HDAC5*). Where indicated, cells were subsequently treated with 25 μM TMP-195. (**A**–**G**) mRNA expression levels of target genes were quantified by qRT-PCR. Each condition was tested in three biological replicates (*n* = 3). (**H**) Schematic summarizing the major findings: *TTN* deficiency suppresses cardioprotective programs and promotes fibrosis, while HDAC5 inhibition with TMP-195 restores functional gene expression and attenuates fibrosis, highlighting therapeutic potential in *TTN*-related cardiomyopathies. Arrows (→) indicate activation, and blunt-ended lines (⊣) indicate inhibition. See main text for details. Statistical analysis: Panels (**A**–**G**) were analyzed using one-way ANOVA with Tukey’s post hoc test. * *p* < 0.05; ** *p* < 0.01; *** *p* < 0.001; **** *p* < 0.0001. Bars represent mean + SEM.

To confirm that the protective effects of TMP-195 are mediated through HDAC5, we co-silenced *TTN* and *HDAC5* in iPSC-CMs, followed by TMP-195 treatment. As expected, neither *HDAC5* silencing nor TMP-195 altered *TTN* expression (Figure 8A), indicating a *TTN*-independent mechanism. For *MYH6*, *HDAC5* knockdown alone restored expression, and TMP-195 did not further enhance this effect (Figure 8B). In contrast, *NPPA* expression was robustly restored by *HDAC5* silencing, and further augmented by TMP195 (Figure 8C), again suggesting a stress-independent epigenetic regulatory mechanism for *NPPA*. For *COL1A1*, *HDAC5* knockdown significantly suppressed its expression, and TMP-195 provided additional suppression (Figure 8D). However, for *COL3A1* and *COL14A1*, *HDAC5* silencing alone was sufficient to reduce expression, and TMP-195 had no additive effect. Notably, *TTN* silencing in iPSC-CMs also reduced *HDAC5* expression, and neither *HDAC5* knockdown nor TMP-195 fully compensated (Figure 8G), suggesting the existence of additional regulatory mechanisms (Figure 8H).

### 3.7. HDAC5 Inhibition Modulates MEF2-Associated Gene Expression

**Figure 9:**
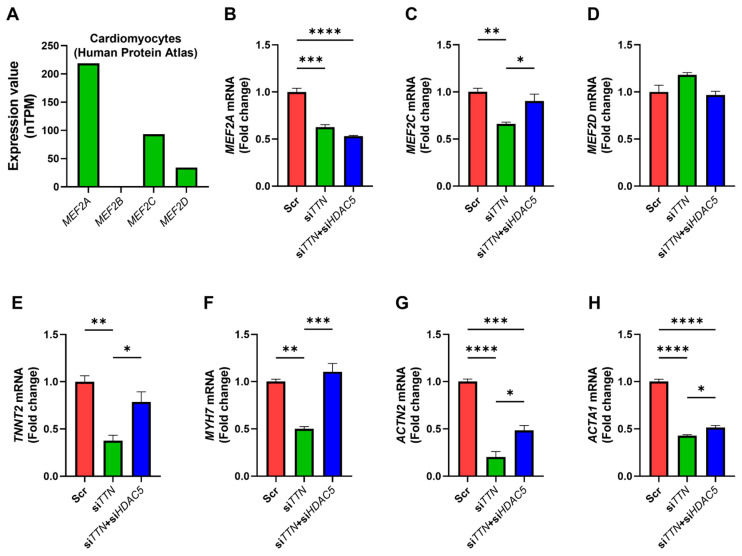
HDAC5 inhibition restores MEF2C-associated gene expression in *TTN*-silenced iPSC-derived cardiomyocytes. (**A**) *MEF2A*, *MEF2B*, *MEF2C*, and *MEF2D* expression levels (normalized transcripts per million, nTPM) across cardiomyocytes, obtained from the Human Protein Atlas. (**B**–**H**) Human iPSC-derived cardiomyocytes were transfected with scrambled control siRNA (Scr), *TTN*-specific siRNA (si*TTN*), or a combination of *TTN* and *HDAC5* siRNAs (si*TTN* + si*HDAC5*). mRNA expression levels of target genes were quantified by qRT-PCR. Each condition was tested in three biological replicates (*n* = 3). Statistical analysis: Panels (**B**–**H**) were analyzed using one-way ANOVA with Tukey’s post hoc test. * *p* < 0.05; ** *p* < 0.01; *** *p* < 0.001; **** *p* < 0.0001. Bars represent mean + SEM.

MEF2 transcription factors, particularly MEF2C, are essential regulators of cardiac development and function, with activity modulated by Class II HDACs [23]. Examination of the Human Protein Atlas (HPA) database, together with our preliminary analyses, revealed that *MEF2A*, *MEF2C*, and *MEF2D* are robustly expressed in human cardiomyocytes, whereas *MEF2B* shows minimal cardiac expression (Figure 9A). In *TTN*-silenced iPSC-CMs, *HDAC5* knockdown was associated with partial restoration of *MEF2C* expression, while *MEF2A* and *MEF2D* remained largely unchanged (Figure 9B–D). Correspondingly, canonical MEF2 target genes linked to myopathy and contractile function, including *TNNT2*, *MYH7*, *ACTN2*, and *ACTA1*, were also restored upon *HDAC5* silencing (Figure 9E–H).

These observations suggest that HDAC5 inhibition may help restore cardioprotective gene programs, potentially through MEF2C-dependent pathways. Although direct regulation of MEF2C by HDAC5 was not conclusively demonstrated in this study, our data provide novel evidence supporting the therapeutic potential of HDAC5 targeting in *TTN* deficiency, warranting further mechanistic investigation.

## 4. Discussion

Our findings demonstrate that alterations in *TTN* expression are associated with various forms of cardiomyopathy, including DCM, HCM, and PPCM, characterized by a notable downregulation of *TTN* expression in DCM patients (Figure 1A–C). This observation aligns with previous genetic studies identifying truncating variants in *TTN* (*TTNtvs*) as the most common genetic cause of heritable DCM [32]. Despite this established role, clinical interpretation of *TTN* variants remains challenging due to the enormous size of the gene, the diversity of its isoforms, and the presence of numerous background variants in the general population [5]. Importantly, our study adds to this complexity by demonstrating that transcriptional dysregulation is not limited to *TTN* itself. We observed significant downregulation of *MYH6*, a gene encoding α-myosin heavy chain and a key determinant of cardiac contractility, across multiple cardiomyopathy subtypes. This suggests that perturbations in *TTN* not only affect sarcomere integrity directly but also initiate broader maladaptive remodeling of the cardiac gene regulatory network (Figure 1D–H and Figure 3). In particular, this is consistent with cardiac stress or remodeling, where loss of *TTN* function triggers fibrosis-related pathways. Recent work also suggests that epigenetic regulation, including DNA methylation changes and alternative splicing of *TTN*, contributes significantly to disease progression in DCM patients [33], underscoring the importance of integrating genetic and epigenetic layers of regulation in *TTN*-mediated cardiomyopathy.

The pathogenic mechanisms of *TTNtv*-mediated DCM are generally explained by two complementary hypotheses: haploinsufficiency and the “poison peptide” effect [9,34]. In the haploinsufficiency model, the loss of one functional allele leads to insufficient production of full-length titin protein, impairing sarcomere assembly and function [6,35]. In contrast, the “poison peptide” hypothesis proposes that truncated titin proteins produced from mutant alleles are incorporated into sarcomere or accumulate intracellularly, where they exert dominant-negative effects [6,35]. Evidence for both mechanisms exists: truncated titin peptides have been detected in diseased hearts and shown to integrate into sarcomeres [36], yet their stability, turnover, potential aggregation, and, most importantly, their relative contribution to disease and the downstream cellular responses such as altered gene expression and proteostasis remain debated [37,38]. Our study contributes to this mechanistic understanding by revealing that *TTN* silencing in iPSC-CMs induces defined changes in gene expression that extend beyond structural protein disruption and are at least partly reversible by targeting histone deacetylases. This suggests that *TTN* deficiency not only destabilizes sarcomere architecture but also perturbs transcriptional and epigenetic programs governing cardiac function (Figure 3).

Epigenetic regulation, characterized by its reversibility and context-specificity, has emerged as an attractive target for therapeutic intervention in heart disease [17]. Our data demonstrate that inhibition of HDAC5 effectively reverses *TTN*-silencing-induced gene dysregulation, thereby identifying HDAC5 as a promising therapeutic target in *TTN*-deficient related cardiomyopathy. These results expand on previous studies exploring histone deacetylase inhibitors as potential treatments for cardiac hypertrophy and fibrosis [17,18,39]. In our experiments, HDAC5 exerted a dual role: it repressed cardiac functional genes such as *MYH6* and *NPPA*, while simultaneously promoting the expression of profibrotic genes including *COL1A1*, *COL3A1*, and *COL14A1*. Pharmacological inhibition of HDAC5 with TMP-195 restored cardioprotective gene expression while selectively suppressing fibrosis-associated genes (Figure 8).

It is important to note that NPPA is a classical stress-responsive and cardioprotective gene, and its upregulation in patient hearts likely reflects systemic pressure overload and remodeling (Figure 1A,B,E) [40]. By contrast, *TTN*-silenced iPSC-CMs are studied in the absence of hemodynamic stress, which may explain the reduced *NPPA* expression observed in vitro. The ability of TMP-195 to restore *NPPA* expression in this context suggests that Class IIa HDACs, including but not limited to HDAC5, contribute to the epigenetic repression of NPPA. Thus, NPPA regulation in cardiomyopathy likely integrates both stress-induced signaling and HDAC-mediated chromatin regulation, highlighting that HDAC5 inhibition may reinforce endogenous cardioprotective responses (Figure 5C, Figure 6C, Figure 7G and Figure 8C).

Of particular interest, TMP-195 generally exhibits a lack of direct overt cytotoxicity across various cell lines, including breast cancer cells and neonatal rat ventricular myocytes (NRVMs) [41,42]. In breast cancer models, TMP195 alone did not affect cell viability, and any observed increase in cell death within tumors was deemed indirect, acting through macrophage modulation [41]. Similarly, in cultured NRVMs, despite elevating reactive oxygen species (ROS) levels, TMP195 did not cause overt cytotoxic effects, possibly due to increased expression of compensatory antioxidant genes [42]. However, TMP195 can enhance drug-induced apoptosis and resensitize multidrug-resistant cancer cells to various cytotoxic anticancer drugs by modulating ABC transporter function [43]. In in vivo studies, no obvious toxicities were observed in mice treated with TMP195 over a 24 h period [44]. These safety observations provide an encouraging foundation for considering TMP-195 in cardiac applications, though long-term cardiotoxicity studies remain essential.

TMP-195 demonstrates significant antifibrotic effects in the kidney. Its mechanism involves inhibiting HDAC9, which in turn alleviates epithelial cell cycle arrest in the G2/M phase [45]. A similar inhibition of HDAC4 by TMP-195 reduces the production of profibrotic cytokines, such as TGF-β1, mitigates tubulointerstitial fibrosis, and inhibits fibroblast activation, thereby offering renoprotection [44]. Taken together, these findings suggest that TMP-195 exerts antifibrotic activity through multiple Class II HDACs, supporting its broader utility in organ fibrosis. In this regard, our results demonstrating the suppression of pro-fibrotic genes like *COL1A1*, *COL3A1*, and *COL14A1* through HDAC5 inhibition are particularly significant (Figure 8), as myocardial fibrosis, characterized by excessive deposition of extracellular matrix proteins (especially collagen I and III), is a critical mechanism in heart failure progression [11]. Interestingly, the antifibrotic effect was not uniform: TMP-195 did not further suppress *COL3A1* and *COL14A1* beyond the effect achieved by *HDAC5* knockdown. This divergence emphasizes that TMP-195 likely acts through both HDAC5-dependent and HDAC5-independent pathways, consistent with its activity against other Class II HDACs. This gene-specific response likely reflects (i) differential chromatin accessibility at individual loci, (ii) compensatory activity of other Class II HDACs, or (iii) engagement of parallel signaling pathways—all of which emphasize the need for isoform-specific mechanistic dissection prior to clinical translation [33]. Overall, these findings indicate that TMP-195, with its favorable safety profile and broad antifibrotic potential, may represent a viable strategy to counteract myocardial fibrosis and functional decline in *TTN*-related cardiomyopathy.

Class II HDACs (HDAC4, 5, 7, and 9) are traditionally viewed as signal-responsive repressors of MEF2 transcription factors, thereby limiting cardiac hypertrophy under basal conditions [20,46]. However, accumulating evidence indicates that their role is context-dependent [23]. Under hypertrophic stress, protein kinases such as calcium–calmodulin-dependent kinases phosphorylate class II HDACs, promoting their nuclear export and thereby releasing MEF2 activity to drive hypertrophic gene expression. Experimental models further reveal a paradoxical role for HDAC5. Its expression is significantly increased in TAC- and Ang II-treated mouse hearts [21,22]. Rather than suppressing hypertrophy, this upregulation enhances hypertrophic and fibrotic remodeling by inhibiting the ERK/EGR1 pathway, which in turn elevates MEF2A expression. Inhibition of HDAC5, either genetically or pharmacologically, has been shown to restore ERK/EGR1 signaling, improve cardiac function, and attenuate remodeling [21]. Similarly, in phenylephrine-treated neonatal rat ventricular myocytes (NRVMs), TMP-195 influences cardiac gene expression by repressing *Xirp2* [42], which is a direct target of the MEF2 transcription factor and plays a role in MEF2A-mediated cardiac remodeling [47]. By contrast, in cardiomyopathy patients, Class II HDACs including HDAC5 expression remain largely unchanged (Figure 4B), implying that this group of HDACs may act in a context- or signal-dependent manner in patients (for example, being pathologically activated without a change in steady-state expression). This discrepancy highlights that findings from experimental models cannot always be directly extrapolated to human pathology and underscores the need for integrative approaches combining model systems with patient data [48].

MEF2 transcription factors exemplify the double-edged nature of cardiac transcriptional regulators. While MEF2 transcription factors are indispensable for cardiac development and function, their overactivation can be maladaptive. Overexpression of MEF2A or MEF2C in the postnatal mouse heart has been shown to induce pathological remodeling, often culminating in DCM [49]. Conversely, under physiological conditions, MEF2s serve as master regulators of muscle gene expression, with MEF2C playing a particularly central role in driving sarcomeric and contractile gene programs, including *Tnnt2* and *Ttn* [24,25]. Other transcription factors such as NKX2-5 also contribute significantly, functioning both independently and cooperatively with MEF2. Indeed, NKX2-5 and MEF2 binding motifs have been identified in a key enhancer region of *TTN*, highlighting their interdependent regulation of this essential gene [50].

A notable observation in our study was that *TTN* silencing in iPSC-CMs reduced *HDAC5* expression, whereas no significant change was detected in DCM patient hearts (Figure 4B and Figure 8G). At first glance, this appears contradictory to our conclusion that HDAC5 inhibition is protective. However, it is important to distinguish HDAC5 expression from its functional activity, which is regulated by phosphorylation and nuclear–cytoplasmic shuttling. Thus, steady-state mRNA levels may not reflect functional repression of MEF2 activity. In vitro, the acute loss of *TTN* may trigger a compensatory downregulation of HDAC5, whereas in the chronic, stressed environment of human myocardium, HDAC5 levels remain stable while its activity may still be pathologically altered. To clarify these mechanisms, future experiments should assess HDAC5 localization and phosphorylation status, measure ERK/EGR1 and MEF2 activity, and test isoform-specific contributions (e.g., HDAC4/7/9) using selective knockdowns. These considerations underscore the complexity of HDAC5 regulation and reinforce the view that therapeutic benefit arises from inhibiting Class IIa HDAC activity rather than altering HDAC5 expression *per se*.

In our iPSC-CM model, *TTN* silencing selectively reduced *MEF2A* and *MEF2C* expression, while *MEF2D* remained largely unaffected. Notably, *HDAC5* knockdown partially restored *MEF2C* levels and reactivated downstream contractile genes, including *TNNT2*, *MYH7*, *ACTN2*, and *ACTA1* (Figure 9). These findings position HDAC5 as a pivotal epigenetic regulator that links *TTN* deficiency to transcriptional repression of MEF2C and its downstream targets. Importantly, this regulation extends beyond contractile function to fibrotic remodeling, underscoring the multifaceted role of HDAC5 in *TTN*-related cardiomyopathies. Nevertheless, the evidence remains suggestive rather than definitive, and additional experiments are needed to establish whether MEF2C reactivation represents a direct consequence of HDAC5 inhibition or occurs through indirect pathways.

This epigenetic influence of HDAC5 on MEF2-associated gene expression provides a potential new dimension to our understanding of maladaptive genetic cardiomyopathies. While our data suggest that HDAC5 inhibition may restore MEF2C expression and re-activate downstream cardioprotective genes, the precise molecular interactions between HDAC5 and MEF2 remain to be fully defined. Nonetheless, these findings point toward an important epigenetic mechanism that could contribute to transcriptional reprogramming in *TTN*-related cardiomyopathies. Furthermore, our use of iPSC-derived cardiomyocytes (iPSC-CMs) highlights their utility as a physiologically relevant model for investigating such pathways. Importantly, epigenetic dysregulation is increasingly recognized as a shared feature of cardiovascular remodeling. For example, Costa et al. [51] reviewed how extracellular matrix remodeling, inflammatory signaling, and smooth muscle cell phenotypic changes drive aneurysm pathophysiology, emphasizing the involvement of epigenetic regulation in vascular remodeling [51]. Thus, placing our findings within this broader framework underscores that epigenetic targeting of HDAC5 may have implications not only for inherited cardiomyopathies but also for cardiovascular remodeling more generally.

In summary, our findings support the therapeutic potential of selective HDAC5 inhibition for *TTN*-related cardiomyopathies. To move toward clinical translation, future studies should (i) delineate molecular interactions among HDAC5, MEF2C, and contractile gene promoters using high-resolution chromatin approaches (e.g., ChIP-seq, CUT&RUN, ATAC-seq), (ii) define the relative contribution of other Class II HDACs and compensatory pathways at specific target loci, and (iii) validate efficacy and safety in in vivo *TTNtv* models and genotype-stratified patient cohorts. Such integrative studies will be essential to bridge mechanistic insights with patient care, ultimately informing precision therapies for inherited cardiomyopathy.

## Figures and Tables

**Table 1:** Primer list.

Gene Name	Forward Primer	Reverse Primer
*TTN*	AGTCCCCATCGCCCATAAGA	TGGAGATTCTTGCTGCTGGAG
*HDAC4*	TGTTTCTGCCTTGCTGGGAA	GAACGGACAGCGTTTGCATT
*HDAC5*	TGCGGAACAAGGAGAAGAGC	GGGCTCCTTTGACTTCGACA
*HDAC7*	GCTTCAATGTCAATGTGGCCT	GCGATGGGCATCACGACTAT
*HDAC9*	TCCAGCCACCCTCATGTTAC	GGGAGACTGAGGATGTAAGGG
*MYH6*	GCCCTTTGACATTCGCACTG	TTCACAGTCACCGTCTTCCC
*MYH7*	CTCCCTGATCCACTATGCCG	CTGATACAAGCCCACGACAGT
*NPPA*	CAGCCCAGCCCAGAGAGATG	AGCTTGCTTTTTAGGAGGGCA
*MYOCD*	GCCATCCTCCAAGCTTCTCT	TGTAAACCAGCCATCTTTTGC
*TNNT2*	GCCCAATGGAGGAGTCCAAA	ATCAGCGCCTGCAACTCATT
*TNNI3*	AGTCACCAAGAACATCACGGA	CCGCTTAAACTTGCCTCGAA
*MYL2*	ATCATGGACCAGAACAGGGA	TCATTTTTCACGTTCACTCGC
*MYL3*	TCCAAGAACAAGGACACAGGC	ATTGCCCTCCTTGTCGAAGA
*ACTN2*	TCTCTTGCTTCTACCACGCT	TCCATTCCAAAAGCTCACTCG
*ACTA1*	ACCTGTATGCCAACAACGTCA	TGATCTTCATGGTGCTGGGT
*MEF2A*	GCAATGCAGGTGGGATGTTG	TGCACCAGTAGCTCCAATCA
*MEF2C*	ACCAGGCAGCAAGAATACGA	TAGCCAATGACTGAGCCGAC
*MEF2D*	CTGGAGGACAAGTACCGACG	CTCTGATTGGACACGGGCAC
*NANOG*	TGGCCGAAGAATAGCAATGG	GGTTCCCAGTCGGGTTCAC
*SOX2*	AGAAGGATAAGTACACGCTGCC	TCATGTGCGCGTAACTGTCC
*POU5F1*	AAACGACCATCTGCCGCTTT	CACGAGGGTTTCTGCTTTGC
*COL1A1*	GCCAAGACGAAGACATCCCA	CGTCATCGCACAACACCTTG
*COL3A1*	TGTTCCACGGAAACACTGGT	GGATTGCCGTAGCTAAACTGA
*COL14A1*	GCTGGGGATGAAAAAGAGATGA	GCCTCTTCTCCAAACATGGC
*RPLP0*	GAAACTCTGCATTCTCGCTTCC	GACTCGTTTGTACCCGTTGATG

## Data Availability

The data analyzed during the current study are available from the corresponding author upon reasonable request.
